# Older Sepsis Survivors Suffer Persistent Disability Burden and Poor Long-Term Survival

**DOI:** 10.1093/geroni/igaa057.454

**Published:** 2020-12-16

**Authors:** Robert Mankowski, Stephen Anton, Gabriela Ghita, Christiaan Leeuwenburgh, Lyle Moldawer, Philip Efron, Scott Brakenridge, Frederick Moore

**Affiliations:** 1 University of Florida, Gainesville, Florida, United States; 2 Aging and Geriatric Research, Gainesville, United States; 3 Biostatistics, Gainesville, United States; 4 Surgery, Gainesville, United States

## Abstract

As in-hospital sepsis mortality has decreased, more “sepsis survivors” are progressing into poorly characterized long-term outcomes. The purpose of this study was to describe the current epidemiology of sepsis in older adults compared to middle-aged and young adults. Design: Prospective longitudinal study with patients categorized into young (≤ 45 years), middle-aged (46-64 years) and older (≥ 65 years) patient groups. 328 sepsis patients were characterized by a) baseline demographics and predisposition factors, b) septic event, c) hospital outcomes and discharge disposition, d) 12-month mortality and e) Zubrod Performance status, physical function and cognitive function at three, six and 12-month follow-up. Follow-up visits were not completed due to death (in 68) and withdrawal of consent (in 32). Compared to young and middle-aged patients, older patients had: 1) significantly more comorbidities at presentation (example chronic renal disease 6% vs 12 % vs 21%), intra-abdominal infections (14% vs 25% vs 37%), septic shock (12% vs 25% vs 36%) and organ dysfunctions, 2) higher 30 day mortality (6% vs 4% vs 17%) and fewer ICU free days (median 25 vs 23 vs 20), 3) more progression into CCI (22%, vs 34% vs 42%) with higher poor disposition discharge to non-home destinations (19% vs 40% vs 62%), 4) worse 12-month mortality (11% vs 14 % vs 33%) and, 5) poorer Zubrod Performance status and objectively-measured physical and cognitive functions with slight improvement over 12 month follow-up. Conclusion: Compared to younger patients, older sepsis survivors suffer with both a higher persistent disability burden and 12-month mortality.

